# Recent Applications of Biopolymers Derived from Fish Industry Waste in Food Packaging

**DOI:** 10.3390/polym13142337

**Published:** 2021-07-16

**Authors:** Francesca Lionetto, Carola Esposito Corcione

**Affiliations:** Department of Engineering for Innovation, University of Salento, Via Arnesano, 73100 Lecce, Italy; carola.corcione@unisalento.it

**Keywords:** fish by-product, fish discard, fish waste valorization, blue economy, sustainable food packaging, circular economy, bioplastic, myofibrillar proteins, active packaging, ocean pollution, fish scales

## Abstract

Fish waste is attracting growing interest as a new raw material for biopolymer production in different application fields, mainly in food packaging, with significant economic and environmental advantages. This review paper summarizes the recent advances in the valorization of fish waste for the preparation of biopolymers for food packaging applications. The issues related to fishery industry waste and fish by-catch and the potential for re-using these by-products in a circular economy approach have been presented in detail. Then, all the biopolymer typologies derived from fish waste with potential applications in food packaging, such as muscle proteins, collagen, gelatin, chitin/chitosan, have been described. For each of them, the recent applications in food packaging, in the last five years, have been overviewed with an emphasis on smart packaging applications. Despite the huge industrial potential of fish industry by-products, most of the reviewed applications are still at lab-scale. Therefore, the technological challenges for a reliable exploitation and recovery of several potentially valuable molecules and the strategies to improve the barrier, mechanical and thermal performance of each kind of biopolymer have been analyzed.

## 1. Introduction

The world fish production in 2019 was estimated to be 177.8 million metric tons and it is expected to expand considerably in the future [1]. As widely recognized by the United Nations’ 2030 Agenda for Sustainable Development and FAO, fishery and aquaculture have an essential role for food security and nutrition [2]. About 70% of fish and seafood is processed before sale, thus producing a huge amount of solid waste deriving from activities such as beheading, de-shelling, degutting, removal of fin and scales, filleting [3], [4,5]. The fish industry by-products generally consist of viscera, muscle tissues, carcasses, heads, fins, skin, scales and bones, being approximately between 50% and 75% of the fresh weight depending on the species [6,7,8,9,10,11]. For example, processing of shrimps and fish fillets generates almost 50% and 75% by weight of waste [3]. About 20% of the fish industry by-products is used as low-value ingredients in animal feed [5,10], but the major part is landfilled or incinerated with consequent environmental, health, and economic damage [12].

Another great issue concerning fishery is given by by-catch, that is, a fish or other marine species that is unintentionally caught. By-catch is either the wrong species, the wrong sex or is undersized or juveniles of the target species. Usually these unintentionally caught animals are not kept, but returned to the sea, a phenomenon called discarding at sea. Discarding constitutes a substantial waste of resources and negatively affects the sustainable exploitation of marine biological resources and marine ecosystems and the financial viability of fisheries [13]. The depletion of oxygen occurs as a result of an increased consumption of aerobic bacteria and other organisms that degrade organic wastes with changes in the benthic environment [14,15]. Fish discarding, in fact, greatly affects the marine ecosystem through the food web. Some scavenger species or seabirds may benefit from discards, influencing their populations and their movements. This environmental impact is so high that the European common fisheries policy aims to reduce the discard practice with obligatory landing [16].

Fish waste represents, hence, a growing issue that urgently need innovative approaches and solutions. To this aim, several projects and measures have been globally employed to prevent food waste [17]. In 2015, the United Nations defined the Sustainable Development Goals to guarantee sustainable consumption and production in order to strongly reduce the global per capita food wastes and to protect marine and maritime environments [16,18]. The need to implement more sustainable practices in the fishery and aquaculture sector necessarily involves the valorization of by-products and discards [19]. Recently, it has been proven that a circular economy approach can be successfully applied to the seafood industry re-using by-products [20]. This approach could be in principle extended also to fishery by-catch contributing to finding eco-friendly solutions for the environmental and economic issues of the planet by preventing the abuse of natural resources. To line up with this green lifestyle, the European Commission approved “Blue Growth”, with the aim to sustain a natural growth in the marine and maritime sectors.

**Figure 1:**
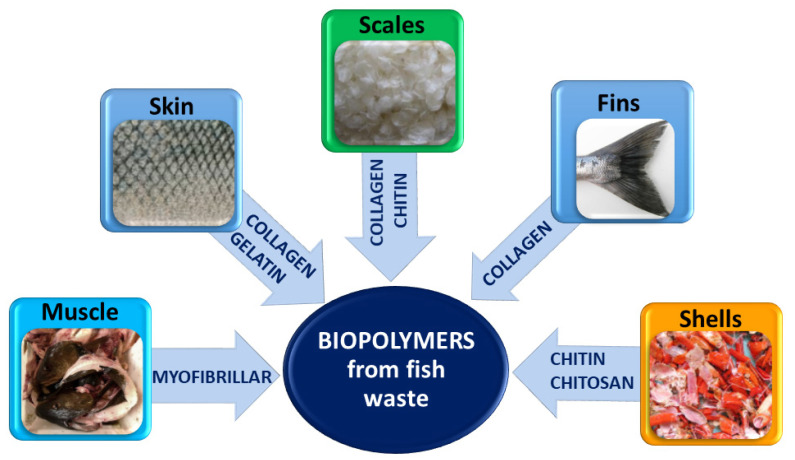
Biopolymers with potential food packaging application derived from fish industry waste.

The valorization of fish waste could contribute to reducing the costs of a safe waste disposal and to generating additional value arising from the recovery of several potentially valuable molecules including oils, proteins, pigments, bio-active peptides, amino acids, collagen, chitin, gelatin, etc. [2,3,10,21,22]. In Figure 1, the different types of fish waste are sketched, e.g., muscle, skin, scales, fins and crustacean shells, together with the derived biopolymers with potential application in food packaging, which will be described in the following paragraphs.

The use of this bio-waste as a raw material, converted into a product with a higher value, leads to the development of biorefinery, now considered a key technology in the 21st century, in contrast to the classical petroleum oil refinery associated with the emission of carbon-based greenhouse gases [23,24]. Polymers derived from fish waste are, in fact, particularly promising substitutes of synthetic polymers for the production of bioplastics [25], which are bio-based or biodegradable, or include both properties [26]. Sometimes, the words “biopolymer” or “bioplastic” have been generally referred to a large variety of plastics with different properties and applications and have been exposed to various significances [2,27]. The bio-based plastics are produced from renewable sources such as starch, cellulose, etc., but some of them can be also not biodegradable such as bio-based-polyethylene [28,29,30,31]. There is general consensus on the potential contribution of biodegradable plastics to the reduction of plastic pollution in the marine environment, which has been recognized as one of the most impacting threats for the environment, causing numerous hazardous and ecologically negative consequences, such as the presence of microplastics and nanoplastics [32].

**Figure 2:**
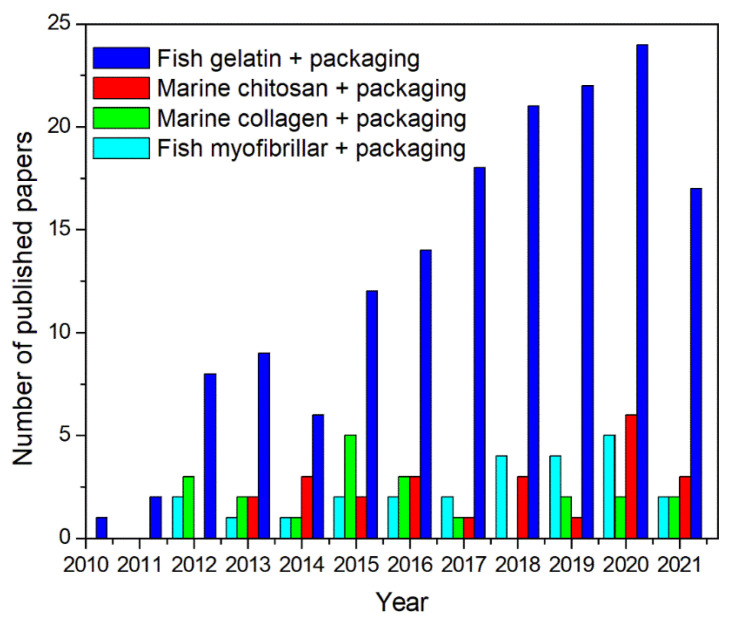
Distribution of scientific papers analyzed by the publication year in the last ten years up to June 2021 (from Scopus database).

During the last decade, the production of bioplastics has largely grown, with the aim of decreasing the negative impact of the synthetic polymers on the environment, since they can be bio-based and/or biodegradable [4,33,34]. However, they still represent a very small segment of the market. In detail, bioplastics are roughly one percent of the more than 368 million tons of plastic supplied each year [35]. Nonetheless, since the market demand is continually growing, including more advanced applications and innovative products, the market for bioplastics is incessantly expanding and differentiating. The global bioplastics production volume is expected to rise from around 2.11 million tons in 2020 to about 2.87 million tons in 2025 [35]. Packaging can still be considered the major market segment for bioplastic production with 47 percent (0.99 million tons) of the total bioplastics market in 2020 [35]. Among these innovative green materials, edible/biodegradable films for food packaging applications have, in fact, recently attracted the attention of both academic and industrial researchers. As reported in Figure 2, the number of scientific papers on this important topic is, hence, largely rising, particularly in the last five years.

This review summarizes the recent advances in the valorization of fish industry waste for the preparation of biopolymers for food packaging applications. In detail, the issues related to fishery industry waste and fish by-catch and the potential for re-using these by-products in a circular economy approach will be presented. Then, all the biopolymer typologies derived from fish waste with potential applications in food packaging, such as muscle proteins, collagen, gelatin, chitin/chitosan, will be described. For each of them, the recent applications in food packaging, in the last five years, will be overviewed with emphasis on smart packaging applications. In addition, the technological challenges for a reliable exploitation and recovery of several potentially valuable molecules and the strategies to improve the barrier, mechanical and thermal performance of each kind of biopolymer will be analyzed.

## 2. Muscle Proteins

**Figure 3:**
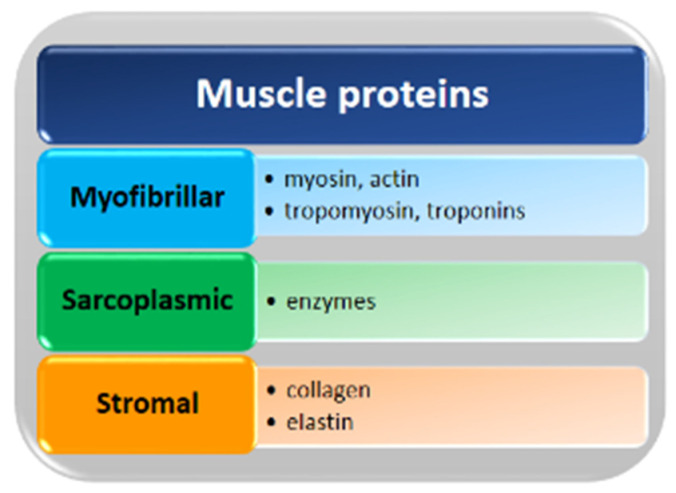
Classification of the principal fish muscle proteins.

As reported in Figure 3, muscle proteins are grouped into three major groups according to their solubility: myofibrillar, sarcoplasmic and stromal proteins. Myofibrillar proteins are the main component of the skeletal muscle representing about 65–75% of the total muscle proteins [36,37]. Myofibrillar proteins include some contractile proteins, such as myosin and actin, regulatory proteins, such as tropomyosin and troponin, and other minor proteins [37]. Due to their structure and localization, myofibrillar proteins require denaturing conditions, e.g., high ionic strength solution to be solubilized and extracted.

Sarcoplasmic proteins are water-soluble proteins, representing about 20–30% of total proteins and mostly consisting of enzymes involved in the biochemical processes of muscle tissues [37]. Stromal proteins, such as collagen and elastin, are present in low content in most commercial fish species and remain insoluble in high-salt solutions [38]. Due to the low extracted content, muscle protein is not the major source of marine collagen, which is more abundant in fish skins and bones [39].

Proteins are one of the most used biomaterials in the food industry due to their nutritional values, non-toxicity, biodegradability and ability to form gels [40]. In recent years, fish stromal and myofibrillar proteins have been receiving significant attention for their ability to form biodegradable edible films with good barrier properties against gases, organic volatiles and lipids [41,42,43,44], which are insoluble in water, but can be made soluble by adjusting the pH of the solution [45]. These films developed from fish myofibrillar or muscle proteins present several advantages: (i) excellent UV light barrier when compared to commercial wrap films made of polyvinyl chloride [46]; (ii) good oxygen and carbon dioxide barrier [12,41]; (iii) slight transparency; (iv) potential for producing active packaging [47].

The major drawback limiting a wide broad commercial application of these films is the rigidity and the low mechanical strength due to the extensive protein–protein chain interactions in the film network that is further reinforced by disulfide bonds, hydrogen bonds and/or electrostatic interactions [44,48]. To overcome this problem, a high content of plasticizers (about 40–60%) is added into the biodegradable film in order to decrease the brittleness and increase the extensibility and toughness by lowering the forces between the protein–protein chains [49]. Another limit of fish myofibrillar protein films is the poor water vapor barrier, due to the high hydrophilic nature of amino acids in the proteins and to the significant amounts of hydrophilic plasticizers added, such as glycerol and sorbitol, to impart the adequate film flexibility [4,15,42]. Chemical crosslinking, electron beam and gamma radiation have been reported as effective methods for obtaining stronger and less permeable films [4,50,51,52].

## 3. Marine Collagen

**Figure 4:**
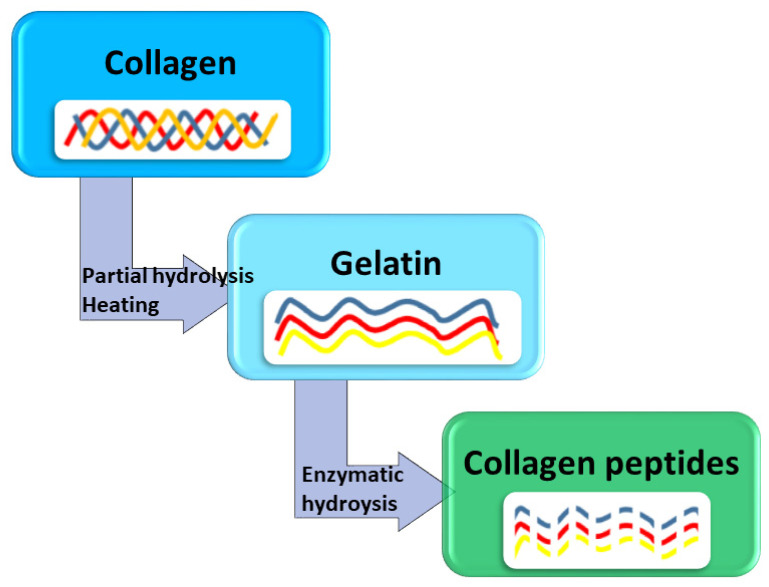
Collagen and its derivatives.

Collagen is the most common animal protein since it is present in all connective tissues (i.e., skin, bones, ligaments, tendons and cartilage) and interstitial tissues of the parenchymal organs [53]. In nature, there are 28 different types of collagen, but the most abundant and plentiful is type I [54], which is also the main constituent of marine collagen. It is characterized by a triple helix structure (Figure 4) made by three cross-linked α amino acid chains, consisting of 2 homologous α1 chains and one α2 chain [39,55]. While the ratio and concentration of amino acids may vary from source to source, structurally, collagen is the same strong molecule regardless of the source.

Marine collagen is mainly extracted from fish skin, bones, fins, scales, or from jellyfish, sea urchin, starfish or sea cucumber connective tissue [56]. Fish skin has been exploited for collagen extraction since about 70–80% of its dry matter is collagen [57,58]. Moreover, another promising and low cost source of marine collagen is given by fish scales [59], representing about 4% of the total weight of the annual output of fish offal, which amounts to circa 18–30 million tons [60,61]. Fish scales contain both organic components (collagen, fat, lecithin, scleroprotein, various vitamins, etc.) and inorganic constituent components (hydroxyapatite, calcium phosphate, etc.) [55,62,63]. The recent and increasing interest towards the valorization of fish scales has led to several applications in environmental protection [64], medicine [65,66,67] and bionics [68].

Compared to mammalian collagen, marine collagen presents a comparable or slightly lower molecular weight and a lower denaturation (melting) temperature [25], which is about 20–35 °C for most fish species with higher values for collagen derived from warm–water species [53,69]. In order to enhance the thermal stability, suitable crosslinking treatments have been studied [70,71].

According to Coppola et al. [53], the yield of collagen extracted from fish byproduct can reach up to higher than 50% in dry mass. Moreover, the oil removal during fish processing guarantees the absence of smell or taste [25]. The extraction of collagen from fish scales by chemical methods often requires a long time. Therefore, the interest of researchers towards suitable processes for extracting fish scale collagen is increasing [30]. For more details on the collagen structure and extraction chemical treatments, readers are addressed to recent literature [2,53,58,69,72].

Compared to mammalian collagen, marine collagen does not present use limitations for religious reasons and for possible transmissible diseases, while having excellent film-forming ability, biocompatibility, low antigenicity, high biodegradability and cell growth potential properties [53,57,73]. This waste material has the potential to be exploited as an eco-friendly and low-cost collagen source with many potential applications in various fields such as health foods, cosmetics and biomedicine as drug/delivery carriers or wound dressings [55,58,74,75,76,77]. Due to its high-water absorption capacity, collagen is a good candidate for texturizing, thickening and gel formation. Moreover, it has interesting properties related to surface behavior, which involves emulsion, foam formation, stabilization, adhesion and cohesion, protective colloid functions and film-forming capacity [53]. Although it is already used as a food additive to improve food rheological properties, marine collagen is yet underexploited, its applications being greatly lower than those with mammalian collagen.

## 4. Fish Gelatin

Gelatin is a denatured protein derived from the partial hydrolysis of collagen followed by thermal treatment (Figure 4) [40,78,79]. It consists of a pool of proteins and polypeptides of different molecular weights, which composition mainly depends on the parent collagen and the extraction procedure [39]. During hydrolysis, the natural molecular bonds between individual collagen strands are broken down leaving a mixture of single or multistranded polypeptides, each with extended left-handed helix conformations and containing 50–1000 amino acids [53,58]. Two types of gelatin, namely, type A and type B, are obtained by acid hydrolysis and alkaline hydrolysis, respectively [53].

Due to religious matters and health concern about the spread of disease to human, the extraction and application of gelatin from fish waste is generating widespread interest [54,80,81,82]. Gelatin is an important industrial biopolymer with significant gelling and film formation properties that make it useful for potential application in food, pharmaceutical and other related fields [58].

Appreciable differences in mechanical and water vapor barrier properties have been reported for gelatin films made from cold-water (cod, salmon or Alaska pollack) and warm-water (tilapia, carp or catfish) fish species, largely as a consequence of differing amino acid compositions. This is mainly due to the amino acid content, which affects the melting point of fish gelatin and thus, the production process [83]. In general, the amino acid content is lower in cold-water fish gelatins than in mammalian gelatins and, thus, these fish gelatins have lower melting points, which could be a benefit in the manufacture of fish gelatin-based products by thermo-mechanical processes due to lower energy consumption and cost, thereby increasing their commercial feasibility [81]. On the other hand, the fish gelatin from warm-water fish could have higher thermal stability, which can be useful in some applications.

The molecular weight distribution, greatly affected by the gelatin manufacturing process, influences the mechanical performance, especially when plasticizers like sorbitol or glycerol are present in the film formulation [83]. More information about the characteristics, the extraction methods and the functional properties of fish gelatin can be found in [84,85,86]. Biodegradability of gelatin-based biopolymer films has led to a growing interest in their use as edible food packaging [87].

Some chemical treatments of gelatin, such as acylation, esterification, deamination, cross-linking, reactions with acids and bases can bring significant changes to its physical and chemical properties [53]. The formation of thermo-reversible gels is obtained by cooling an aqueous solution of gelatin with a content higher than 0.5% to approximately 35–40 °C. The rigidity or strength of the gel depends upon gelatin concentration, structure and molecular mass, pH, temperature and presence of any additives [88]. Generally, collagen and gelatin are processed by wet (or solvent) process, based on the dispersion or solubilization of collagen and gelatin in a solvent, the deposition by film casting, compression molding, extrusion, etc., and the solvent removal by drying or through a solvent–non solvent exchange mechanism [53,58]. In order to control the rheological properties during processing and improve the final properties, especially the deformability, usually one or a blend of plasticizers are used.

## 5. Chitin and Chitosan

**Figure 5:**
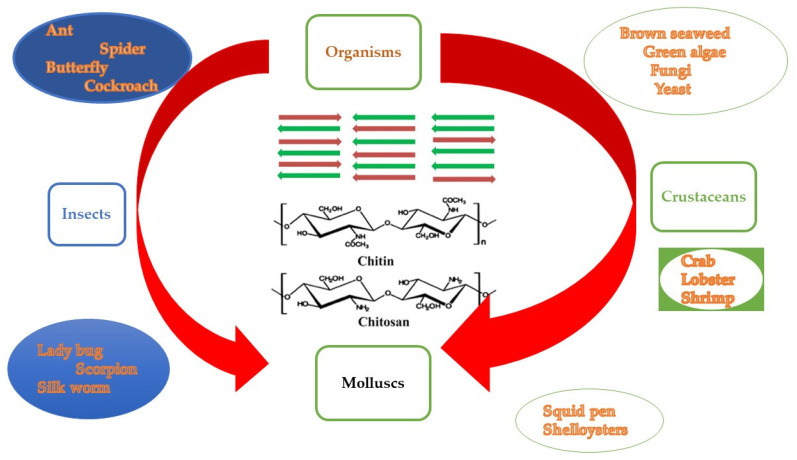
Sources of chitin/chitosan.

Chitin, the second most abundant biopolymer in nature after cellulose, is a linear polymer, i.e., a polysaccharide, sited in fungi cell walls and plankton, in crustacean and insect exoskeletons (Figure 5), under the form of ordered crystalline microfibrils [21]. These organisms produce about 100 billion tons of chitin each year [89]. The chemical structure of chitin, reported in Figure 5, is different from that of other sugars, since it is characterized by the presence of nitrogen [90]. It appears as a yellowish powder, with a high molecular weight, insoluble in water and organic solvents, composed of N-acetyl-2-amino-2-deoxy-D-glucose units joined together by glycosidic bonds β, forming a linear chain with some of the deacetylated monomer units (Figure 5) [91,92]. Chitin can be found in nature in three crystalline structures, α, β and γ (Figure 5), which differ by the number of chains per cell, degree of hydration and unit size [89].

The first experimental studies on chitin isolation started in 1811 by Henri Braconnot, who exposed a variety of fungal species to an aqueous alkali solution, making, thus, available the extraction of the fungine [89]. Since a significant part of environmental pollution is produced by fishing industries wastes, characterized by a distasteful odor, responsible for attracting and accelerating the proliferation of insects, the valorization of chitin extracted form marine wastes could represent an ambitious academic and industrial goal [93,94]. It is well known, in fact, that the fishing industry effluents cause physical and chemical changes in water bodies, with dramatic consequences for aquatic animals (such as a great increase of their mortality), influencing, in turn, the local microfauna and microflora. However, owing to its insoluble nature, the possibility to extract chitin from natural organism, in order to re-use it as a biopolymer, was mostly discounted until recently, when its extraction from shrimp, crab and lobster shells, at industrial or semi-industrial scale, is becoming easily possible, leading to evident advantages, related to the abundance of this material as a derivative of the shellfish managing business [95]. The most used chitin extraction methods are: chemical extraction, chemical deproteinization, chemical demineralization, discoloration, biological extraction, enzymatic deproteinization and fermentation. Chemical extraction uses a strong alkaline solution, in order to obtain the breakdown of polymeric chains, reaching, hence, a high degree of chitosan deacetylation [2,96]. Deproteinization implies the disruption of chemical bonds between proteins and chitin, by using chemical substances to depolymerize the biopolymer [97]. Demineralization uses strong acids, such as sulfuric acid, hydrochloric acid, acetic acid, nitric acid and formic acid, in order to remove minerals, mainly calcium carbonate [98,99,100,101]. Discoloration is an additional step during the extraction process useful to obtain colorless products, by eliminating astaxanthin and carotene pigments from the extraction source, by means of organic or inorganic solvents (acetone, sodium hypochlorite and hydrogen peroxide) [102]. Biological extraction is a more economic, cleaner and greener process consisting in the use of microorganisms to obtain enzymes and organic acids, obtaining, in turn, high quality chitin [103]. Enzymatic deproteinization involves the addition of enzymes for protein fragmentation, with a consequent advantageous absence of environmental degradation sub-products [104,105]. Fermentation allows the productions of hydrolyzed proteins, useful in the food industry, starting from proteolytic enzymes obtained by the lactic acid bacteria, stimulated in a low pH medium [106]. Recently, crustacean wastes of the fishing industry have been successfully used to extract commercial chitin. The major sources are shrimp, crab, lobster, prawn and krill shells. These wastes typically include chitin (20–30%), a protein fraction (30–40%), inorganic salts, mostly calcium carbonate and phosphate (30–50%), and lipids (0–14%) [107]. Moreover, fish scales are also a potential source of chitin, as recently demonstrated [108,109].

The most important byproduct of chitin is chitosan, whose main advantage, compared to chitin, is its solubility. Chitosan, in fact, can be much easier solubilized in dilute acidic aqueous solutions, thanks to the presence of free amino groups in its chemical structure (Figure 5) [89]. Furthermore, the solubilization of chitosan allows one to easily convert it into hydrogels, 3D porous scaffolds, membranes and films, all characterized by good mechanical strength and permeability properties. Chitosan is a polysaccharide, obtained by chitin deacetylation reaction through alkaline hydrolysis and successive treatment with 2-amino-2-deoxy-D-glycopyranose units, merged by glycosidic bonds. It usually has a degree of deacetylation of about 50% or even more. The structural properties of chitosan, as, for instance, the degree of deacetylation and the molecular weight, associated with its physicochemical and biological properties, depend on the relative proportions of 2-amino-2-deoxy-D-glycopyranose units [110,111,112]. Chitosan was discovered in 1859 by treating chitin with heated potassium hydroxide and it is broadly employed in agriculture, medicine, food, cosmetic and textile applications, as a biomaterial, because of its biocompatibility, biodegradability and absence of toxicity. It also worked as a therapeutic agent thanks to its antibacterial and antifungal properties [113,114]. However, its potential utilization could certainly be much larger, including for example tissue engineering, drug delivery, wound dressing, scaffolds, pharmaceutical contaminant removal, cancer diagnosis, composites and nanocomposites, high-tech materials, packaging, dye removal. In nanocomposite material preparation, chitosan has been demonstrated to be successful as a stabilizing agent, for example, for titania nanoparticle dispersion [115], expanded graphite [116], graphene oxide [117,118] and rare-earth elements [119]. In addition, both chitin and chitosan can be mixed with other biopolymers, such as poly (vinyl alcohol), alginate, collagen, cellulose acetate, by obtaining bio-polymer blends, characterized by improved mechanical properties [120,121,122,123]. On the other hand, differently from chitin, thanks to its solubility in both water media and acid solutions, chitosan can be produced in various forms (particles, films, sponges, membranes, gels, fibers) [124]. In particular, chitosan-based films, also thanks to the addition of graphene nanoplatelets, allow one to decrease the permeability of moisture, confirming their potential applications as packaging films for food, with evident advantages related to the re-use of dangerous wastes [125], [126]. Chitosan was also used as an edible antimicrobial coating of rainbow trout for storage at 4 °C for 16 days [127], or as supporting material for the production of antimicrobial coatings for fresh Indian salmon (Eleutheronema tetradactylum) fillets [128].

## 6. Technological Properties of Biopolymers Derived from Fish Waste for Food Packaging

Food packaging is expected to preserve and protect of all types of foods, mainly from oxidative and microbial degeneration, thus increasing their shelf-life [129]. Beside the traditional function of preservation, information and marketing, nowadays food packaging should possess additional functionalities improving their barrier, mechanical and durability properties [130]. Biopolymers derived from fish waste have the potential to overcome the traditional passive role of packaging leading to the development of smart packaging. This new generation of packaging involves interactions between packaging and food or internal packaging atmosphere. Smart packaging comprises both active and intelligent packaging. Active packaging actively interacts with food and packaging headspace to extend the shelf life of food maintaining nutritional and sensor quality and microbial safety [115,116]. Intelligent packaging actively monitors and reports on product conditions and history [131]. The active packaging developed starting from fishery by-products can be in the form of edible coatings or film. Edible coatings are applied to foods by spraying or dipping while edible films are produced separately by solution castings or compression molding and then applied to food surface, by coating, wrapping or spraying, [8,132]. Their aim is to prevent the migration of moisture, oxygen, carbonic dioxide, aromas and lipids, to transport ingredients or bioactive compounds (e.g., antioxidants, antimicrobials and flavor), and/or to improve the mechanical integrity or handling characteristics of the food [18,38].

**Figure 6:**
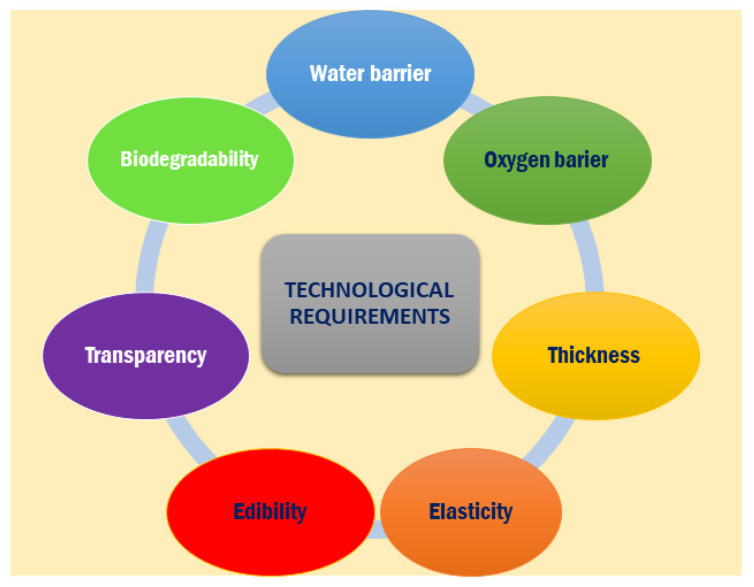
Technological requirements of biopolymers derived from fish by-products for food packaging.

Among the technological requirements of biopolymers derived from fish by-products for food packaging summarized in Figure 6, biodegradability is very important since the use of synthetic packaging films has led to worrying environmental complications. Thus, the utilization of proper bio based and biodegradable packaging films is nowadays becoming a crucial issue and there is an increasing awareness towards packaging made in accordance with the principles of sustainable development [133].

The water vapor permeability (WVP) is one of the most important properties of a packaging material to prevent food drying. It depends both on film porosity and permeability, which is also dependent on temperature, pressure and humidity. The WVP level depends on the specific applications since, for example, dry food or fruits and vegetables needs film with very low and moderate WVP, respectively, to prevent moisture uptake from the environment.

Films and coatings should possess a high oxygen barrier to control oxygen exchange between food and the surrounding atmosphere, protecting food and postponing its degradation by discoloration or surface softening [81]. Referring to oxygen barrier properties, it is well known that, unfortunately, the oxidation of highly unsaturated food lipids, such as fish and seafood, causes dramatic food quality worsening, with consequent off-odors, off-flavors, nutrition losses and color or textural declining [81]. Since film thickness generally influences the final performances of films (mechanical, water vapor permeability, light transmission, transparency), the possibility to control this aspect is decisive in order to produce suitable films for food applications [46]. Further properties are required for packaging materials in direct contact with food, such as adequate sensory properties, biochemical, physicochemical and microbial stability, the absence of toxics, and safety. These necessities are completely satisfied by natural polymers (generally prepared from solutions containing biopolymer, plasticizer and solvent), thanks to their biodegradability and environmental compatibility. Nevertheless, their mechanical properties and permeability are still often lower than those synthetic polymeric materials. Consequently, an important rising interest of food industries is the possibility to use proper biodegradable packaging films possessing not only high thermal and barrier properties, but also outstanding mechanical properties, able, therefore, to increase the shell life of the food products, by protecting them from any kind of undesirable contamination. To this aim, cheap, renewable and largely accessible biopolymers are nowadays generally proposed as a green option over petrochemical polymers [134,135]. Several researchers are studying the possibility to develop biopolymers able to satisfy the most important requirements for food packaging applications, such as optical, barrier and mechanical properties [81].

Antimicrobial and/or antioxidant functionalities in food packaging aim to kill or suppress microbial growth and delay the oxidation of pigments and lipids present in food by incorporating active agents into the packaging materials [114,136]. This active functionality in packaging can help in reducing the economic loss associated with food spoilage. Concerning optical properties, the transparency and the gloss of packaging films are almost mandatory in order to enhance the product appearance and, thus, increase the customer satisfaction [81].

## 7. Applications in Food Packaging

### 7.1. Applications of Biopolymers from Muscle Proteins in Food Packaging

Biopolymers obtained from muscle proteins show a high potential for food packaging applications, mostly as edible films that may be wrapped, coated or sprayed over foods. These films act as a selective barrier against the transmission of gases and vapors, thus improving food quality and extending shelf life. In addition, these fish protein films may have functional properties thanks to the incorporation of functional components like antioxidants, vitamins and coloring agents [3]. The properties of bio-based and biodegradable polymer films derived from muscle proteins reported in the literature are summarized in Table 1. Moreover, the traditional technologies used for thermoplastic polymers can be applied also to these biopolymers, as firstly demonstrated by Cuq et al. [137]. The addition of plant extracts can provide antioxidant and antimicrobial activity as shown by Kaewprachu et al. [138] on fish myofibrillar protein films with catechin–Kradon extracts. The same authors investigated also the effects of various plasticizers on the film properties [48]. The film plasticized with sorbitol exhibited the highest tensile strength (12.56 MPa) and film solubility (62.6%) but, in general, all the fish myofibrillar protein films presented lower strength and flexibility than the commercial PVC films [48].

Romani et al. [139] obtained stiff gels by conformational changes in the structure of fish protein through pH changes. Zavareze et al. [41] prepared biodegradable films from fish myofibrillar and residue protein isolate from Whitemouth croaker achieving low water vapor permeability and a tensile strength close to the values reported by García et al. [140] in *Nile tilapia* muscle protein films. Nie et al. [44] prepared edible/biodegradable films made of silver carp myofibrillar proteins and tannins (tannic acid and apple procyanidins) at various concentrations under alkaline and heating conditions. They used natural phenolic compounds as a crosslinking agent to enhance the mechanical strength, water resistance and thermal stability of the film. Gautam et al. [3] produced a film from a mixture of fish proteins, glycerol and antioxidants. Some films were prepared also with the addition of starch.

Araujo et al. [12] produced bioplastics from myofibrillar proteins from gilded catfish (*Brachyplatystoma rousseauxii*) waste. A response surface methodology was employed to optimize the process design, obtaining a bioplastic with 40% plasticizer (m/m) and 0.79% protein (m/v), characterized by flexibility, mechanical strength, low solubility and water vapor permeability which made the material suitable for food packaging. The good tensile strength (4.91 MPa) was ascribed to the extent of sulfhydryl groups at the myofibrillar protein surface which enabled the formation of covalent S–S in the biofilm framework. On the other hand, the hydrophilicity of fish muscle proteins due to their content of polar amino acids and hydroxyl (OH) groups was responsible for the low moisture barrier of the bioplastic.

The film stability during storage is also an important factor in food packaging, as studied by Leerahawong et al. [141] on mantle-muscle films from *Todarodes pacificus*. The water vapor permeability remained relatively constant during the study while tensile strength increased significantly during the first 10 days, likely due to protein crosslinking caused by the Maillard reaction, while no changes were observed in the elongation at break.

A promising strategy to improve mechanical properties consists in the addition of gelatin and plasticizer into fish protein films, thus diminishing brittleness and improving mechanical properties [42]. The plasticizer should be kept as low as possible in order to avoid excessive hydrophilicity of the film [42,142,143]. As demonstrated by Neves et al. [144], the mixture of gelatin and myofibrillar fish proteins can improve the technological properties of the biodegradable film, making its application feasible in food packaging [144].

**Table 1:** Properties of bio-based and biodegradable polymer films derived from muscle proteins.

Fish Waste Source (Starting Material)	Protein Type and Content (%)	T (mm)	TS (MPa)	EAB (%)	WVP (× 10^−11^ g m^−1^ s^−1^ Pa^−1^)	S (%)	Ref.
**Whitemouth croaker**	myofibrillar	0.132	5.41	251	2.5	31	[41]
**Yellow stripe trevally**	protein isolate/gelatin blend	0.036	13.98	64	3.3	42	[42]
**Argentine anchovy**	protein isolate	0.113	0.6	28	11.6	45	[45]
**King weakfish**	myofibrillar/gelatin blend	0.106	6.5	384	2.7	27	[144]
**Gilded catfish**	myofibrillar proteins	0.033	4.9	178	6.4	19	[12]
**Sardine**	proteins from bones, heads, guts, and fins		0.21	0.34	-	-	[145]
**Tilapia**	myofibrillar protein/sorbitol	0.014	12.5	66	3.0	63	[48]
**Catfish**		0.17	1.27	88	7.7	15	[4]
**Silver carp**	myofibrillar/glycerol/tannic acid	0.06	3.9	94	15	2	[44]
**Whitemouth croaker**	10%	0.114	4.2	28	8.6	100	[139]
**PVC film**	-	0.010	46.9	268	3.1	-	[46]

T: Thickness; TS = Tensile strength; EAB: elongation at break; WVP: water vapor permeability, S: solubility.

### 7.2. Applications of Marine Collagen in Food Packaging

Marine collagen films and coatings are finding increasing application in the food packaging development of sustainable packaging materials to protect, maintain and extend the shelf life of foods, mainly as integral/edible parts of food products [75,115]. Generally, food-packaging materials are required to act as a barrier against the migration of oxygen and moisture, as well as to preserve the sensory qualities and prevent fat oxidation, discoloration and microbial activity. The best known industrial application of collagen consists in edible casings for meat processing industries (sausages/salami/snack sticks) that are able to shrink and stretch to accommodate contraction and expansion of meat batter during continuous processing [57]. The preparation of collagen films is generally achieved by using a plasticizer, mainly glycerol in the range 20–30 wt%, a small molecule of low volatility added to decrease attractive intermolecular forces along polymer chains and increase free volume and chain mobility. Ahmad et al. [57] prepared collagen films obtained from the skin of starry triggerfish based on acid solubilized or pepsin solubilized collagen. This latter had higher thermal stability and mechanical properties with a smoother and homogenous surface compared to the films obtained from acid solubilized collagen, as reported in Table 2.

The use of fish collagen films is limited in the packaging industry by some disadvantages such as low thermal stability and relatively poor mechanical properties [146,147]. Moreover, collagen is a hydrophilic polymer which has hydroxyl groups; thus, the water vapor could easily permeate through the film. To overcome these limitations, various efforts are made, including the blending of collagen with other biopolymers and several chemical and enzymatic treatments. For example, Ahmad et al. [147] used a blend of collagen extracted from unicorn leatherjacket skin and chitosan, which enhanced the film bacteriostatic capacity and fungistatic activity but affected the film elasticity or brittleness. The same authors developed composites films through a blend of the same collagen with soy protein isolate, which is an amphiphilic molecule obtained as a highly refined by-product of soybean oil industry and represents a promising alternative for synthetic polymers [147]. Owing to its non-cytotoxicity, abundance in nature, low cost, nutritive value and hydrophobicity, the blend of soy protein isolate with collagen showed enhanced water vapor barrier property, as reported in Table 2. Wang et al. [129] prepared collagen films with sodium alginate, using glutaraldehyde as a cross-linking agent. According to the authors, the hydrogen and electrostatic interactions between carboxylate groups of sodium alginate and hydroxyl groups of collagen lead to a dense matrix with improved thermal stability and mechanical strength and reduced water vapor permeability. The properties of some bio-based and biodegradable polymer films derived from marine collagen reported in the literature are summarized in Table 2.

**Table 2:** Properties of bio-based and biodegradable polymer films derived from marine collagen.

Fish Waste Source	T (mm)	TS (MPa)	EAB (%)	WVP (g m^−1^ s^−1^ Pa^−1^)	S (%)	Ref.
**Starry triggerfish *A. stellatus* (skin)**						[57]
**acid solubilized**	29	47	28	4.8 × 10^−10^		
**pepsin solubilized**	29	34	40	6.6 × 10^−10^		
**Unicorn leatherjacket *Aluterus Monoceros* (skin)**	21	25	15	3.0 × 10^−10^		[147]
**Blend with chitosan CG/CH (8:2)**	31	20	24	4.5 × 10^−10^		
**Blend with soy protein isolate CG/SPI (8:2)**	28	40	8	2.4 × 10^−10^		
**Smooth-hound** ***Mustelus mustelus* (skin)** **Collagen-chitosan film 25:75**	16	66	4		18	[148]
**Fish skin collagen** **(Shanghai Yuanye Bio-Technology Co)** **Collagen/sodium alginate (10:2)**	32	26	65	1.7 × 10^−10^		[129]

T: Thickness; TS = Tensile strength; EAB: elongation at break; WVP: water vapor permeability, S: solubility.

### 7.3. Applications of Fish Gelatin in Food Packaging

Thanks to good film-forming properties, low cost, biocompatibility and biodegradability, fish gelatin has been recently recommended for the preparation of biodegradable films in active food packaging, replacing conventional non-biodegradable polymers and other mammalian-based gelatins [149,150]. Gelatin is easily processed by applying heat and mechanical stress in extrusion-based technologies [53]. In order to increase the flexibility, a plasticizer is used as an internal lubricant, leading to increased molecular mobility [79]. Gelatin films can be obtained through casting from the gelatin aqueous solution. They are tasteless, colorless, transparent, water-soluble and present higher flexibility properties than other bio-based films for food packaging [149]. Since the melting point of gelatin is close to body temperature, the gelatin-based films can be used for the preparation of edible films [132,150]. In addition, fish gelatin has shown great potential as an excellent matrix to host bio-active compounds with enhanced functionalities, such as antioxidant/antimicrobial [149].

**Figure 7:**
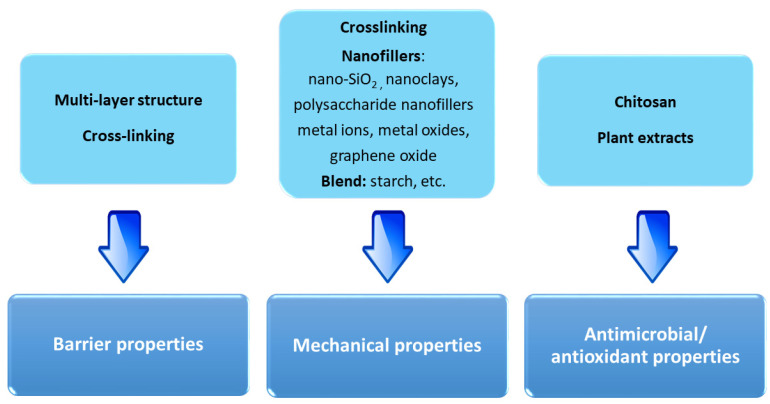
Strategies for improving the performance of fish gelatin and/or chitosan films for food packaging.

The use of fish gelatin films in food packaging is limited by some drawbacks, such as the high hygroscopicity, which is responsible for a drastic reduction in moisture barrier and mechanical strength [53,151], and low oxygen permeability [150]. To overcome this weakness, some successful strategies summarized in Figure 7 have been recently investigated.

The water barrier properties have been improved by laminating fish gelatin films with moisture resistant biodegradable polymers in a multi-layer film with optimized moisture and oxygen barriers for specific package and conditions [53]. Martucci et al. [152] obtained a three-layer gelatin film by hot compression of sodium montmorillonite-plasticized gelatin as the inner layer and cross-linked dialdehyde starch and plasticized gelatin films as the outer layers. The multilayer film displayed a compact and uniform microstructure due to the highly compatible individual layers which could interact by strong hydrogen bonding. The same authors prepared also a multi-layer structure with poly (lactic acid) films as outer layers achieving a water vapor permeability higher than that obtained from other commercial polymers such as high density polyethylene or poly (vinyl chloride) [153].

Another promising approach to improve the barrier, mechanical and thermal properties of fish gelatin for food packaging is based on crosslinking [154,155]. In particular, natural based crosslinking agents have attracted more attention in order to take into account environmental and health concerns, along with the economic issues, as reviewed by Garavand et al. [155]. Liguori et al. [156] have developed a protocol for crosslinking fish gelatin with citric acid. Heat treatments in the presence of reducing sugars, known as the Maillard reaction, have been demonstrated to lead to a crosslinking process and modified network structure [157]. Very recently, Maroufi et al. [158] demonstrated the chemical crosslinking of fish gelatin with the aldehyde groups of K-carrageenan.

A popular and attracting strategy for the realization of active packaging from fish gelatin is based on the reinforcing with different types of nanofillers [86] such as nano-SiO_2_ particles [159], nanoclays (montmorillonite, sepiolite, halloysite) [160,161], polysaccharide nanofillers (nanowhiskers, nanofibers, micro and nanocrystalline cellulose) [162,163,164,165,166,167] metal ions like silver, copper, etc., metal oxides nanoparticles such as ZnO [168,169] or TiO_2_ [170]), coconut husk [171], or chitosan nanoparticles [172,173]. The data reported in the literature confirm the improvement of their performance in food packaging systems thanks to the large interfacial area between the nanofiller and the biopolymer matrix [174].

The hydrophilic character of graphene oxide leads to strong physical bonds with hydrophilic polymers like gelatin with a consequent good compatibility [175]. More recently, Adilah et al. [176] produced nanocomposite fish gelatin from Tilapia fish skin with graphene oxide (up to 2% by weight) characterized by improved barrier and mechanical properties compared to unfilled gelatin. Film properties can be enhanced by adding also proteins (soy protein isolate), oils (sunflower oil, fatty acids, essential oils) [177], pectin [178], starch [179,180] and cross-linkers (glutaraldehyde, MTGase, EDC) in order to improve the rheological properties, barrier properties and water resistance of composite fish–gelatin films [83]. Moreover, antioxidants can be added to the film formulation leading to better food preservation. Very recently, a high number of studies is focusing on the use of natural antioxidants from plant extracts. For example, olive extracts [181], orange [178], fruit berries [182].

The gelatin extracted from fish scales has been widely used in encapsulation and edible film formation [79]. Azmi et al. [79] investigated the functional properties of Tilapia’s fish scale gelatin films with various type of plasticizers. They found that the addition of plasticizers with different hygroscopicities affected the glass transition temperature, the thermal degradation, the chemical interaction between protein and plasticizer, the strength and flexibility of the plasticized films. Weng and Wu [54] prepared edible films based on tilapia scale gelatin with improved thermal stability and mechanical properties thanks to thermal treatments at temperature between 100 °C and 120 °C which promoted the cross-linking in the gelatin film network between β-chain and α-chains. The main interactions involved in the gelatin film formation changed from ionic and hydrogen bonds to hydrophobic interactions and covalent bonds, thus improving the water resistance of the films.

Gomez-Estaca et al. showed that the application of chitosan–gelatin film can delay or even inhibit the growing of microorganisms on fish, suggesting their suitability for fish protection [136]. Chitosan–gelatin protective films have also been proven to be suitable in the preservation of the shelf life of rainbow trout and Pacific white shrimp, kept in refrigerated environments [183,184]. The positive effects of chitosan–gelatin coatings led to both oxidation and spoilage reduction, increasing food shelf-life, demonstrating, in turn, their availability for the specific application. However, the impacts of these products in terms of toxicological effects during handling or consumption still need consideration [134].

### 7.4. Applications of Chitosan in Food Packaging

The biocompatibility, nontoxic and biofunctional properties of chitin and chitosan biopolymers make them potentially suitable for food packaging applications [185,186,187]. In particular, chitosan biopolymer, extracted form shrimp, was preconized as Generally Recognized As Safe (GRAS) [188,189]. On the other hand, chitosan is significantly cheaper in comparison to other biopolymers. Nonetheless, the outstanding properties of chitosan make it a greater candidate for food packaging applications. As an example, it was successfully proposed for increasing the shelf life of bread, since it was demonstrated that it is able to delay the starch retrogradation by preventing the microbial growing [190,191].

Tyliszczak et al. [192] demonstrated that chitosan films allow a strawberry preservation, too. Furthermore, in [193], Zakaria et al. evidenced that chitosan films inhibit alterations in the physical properties of vegetables. Chitosan can be also used for the production of paper for food packaging coated with it, thus delaying the microbial growth [193]. The strategies for improving the performance of chitosan films for food packaging (Figure 6) are nearly the same as adopted for fish gelatin films. In fact, the development of polymer blends represents a valid approach to enhance mechanical performances and reduce the water solubility and the water vapor permeability [194,195]. Several polysaccharides have been added to chitosan for producing blended films with enhanced final properties for food applications. Among them, thanks to its low cost, wide availability and biodegradability, starch is one of the most common polysaccharides proposed for the production of chitosan-based biofilms [196,197].

Chitosan/starch films showed reduced bacterial adhesion on the packaging, excellent antioxidant activity and increased water vapor barrier properties, demonstrating, thus, their potential suitability for the specific proposed application [198,199]. Several scientists studied the possibility to prepare cellulose/chitosan blends in order to improve mechanical properties of the neat chitosan [200]. As an example, Youssef et al. showed that chitosan/carboxymethyl cellulose films were able to enhance the shelf life of cheese and wheat bread [201,202]. In order to increase mechanical and barrier properties of chitosan films for food packaging, several nanoparticles (such as graphene or carbon nanotubes, silver nanoparticles) have been also added to the biopolymer, obtaining a different kind of nanocomposite. In [125], the addition of nanometric graphene stacks to the cinnamaldehyde-functionalized chitosan films was evaluated with the aim to increase the mechanical properties of the films. The nanocomposite films were also tested for antifungal properties with bread slices against a selected mold line, showing a greater activity compared to the biopolymer without nanofiller. Silver nanoparticles, with antimicrobial activities against a large range of pathogenic microorganisms, have been also incorporated into chitosan films for food packaging, allowing a great increase of the antibacterial activity, hydrophilic property, degradability, biocompatibility and nontoxicity of the biofilms [203]. At the same time, the addition of extracts from plants to chitosan appreciably improves the film properties, such as antimicrobial and antioxidant activity, barrier mechanical and thermal properties, obtaining, in turn, a synergistic effect between chitosan and plant extracts [203]. Furthermore, several proteins, achieved from plants, animals or microorganisms, have been added to chitosan to form films with different final properties, which encourage their application in food packaging. As an example, chitosan/caseinate films exhibited increased water vapor permeability [204]. Chitosan/collagen blends showed higher thermal stability, good adhesion, and compatibility [147]. Lysozyme−chitosan films enhanced the freshness of the egg during storage, improving the shelf-file of the product [205]. As also reported for fish gelatin films, for chitosan based films, the crosslinking with different methods has been reported as a viable strategy for improving the performance [155].

Finally, an emerging research area is focused on chitosan nanoparticles as green fillers, for the reinforcement of various biodegradable composites for food packaging and biomedical applications [206,207]. It is reported that the addition of chitosan nanoparticles to biocomposites can significantly enhance their thermal, physical, mechanical, antimicrobial and structural features.

### 7.5. Applications of Biopolymers from Fish Scales in Food Packaging

The mixing of fish scales with biopolymers to enhance the performance and applicability of this renewable resource has been recently reported. Thammahiwes [67] used fish scale wastes as a bio-filler for preparing green composites with wheat gluten characterized by an increased tensile strength. Chiarathanakrit et al. [208] demonstrated that the addition of calcinated fish scales increased the tensile strength of wheat gluten-based bioplastics and starch foams for replacing polystyrene based packaging. Nourbakhsh et al. [209] reported that fish scale waste could increase the biodegradation rate of polypropylene.

Microbial fermentation of scales, consisting in the break of carbohydrates by microorganisms, is being studied with the aim of producing sterilized bioplastics without any residual odor [87]. Moreover, several household goods have been manufactured from bioplastics derived from fish scales. As an example, the designer Erik De Laurens patented a plastic material made only from fish scales, heat and pressure treated, with which he produced a pair of swimming goggles, spectacles and beakers [210].

An interesting application is MarinaTex, a biodegradable polymer derived from the combination of fish waste and red algae patented by Lucy Hughes [211]. This product, in the form of a transparent film, decomposes in six weeks at lower temperatures compared to other bioplastics, which can be reached in a home compost bin. According to its inventor, Lucy Hughes, the film has a higher mechanical strength than low density polyethylene (LDPE) film of the same thickness. This promising material still needs a successive development for a mass production.

## 8. Conclusions and Future Perspectives

Fish industry waste is demonstrating its great potential as a new raw material for biopolymer production in different application fields, mainly in food packaging. The valorization of fish waste presents economic advantages since it could contribute to reducing the costs of a safe waste disposal an generate additional value arising from the recovery of several potentially valuable molecules. Moreover, the valorization of fish by-products presents several environmental advantages arising from the reduction of landfilling, incineration and discarding, which constitute a substantial waste of resources, and from the replacement of fossil-based polymers. In this way, the recovery of fish industry waste could positively affect the ecosystems and the financial viability of fisheries. Its diffusion is expected to increase in the next years, in particular in developing countries, and can contribute to alleviate the waste accumulation problem due to petrochemical derived plastics.

The main contribute of this review is to show that all fish industry by-products can be potentially exploited for the development of the biorefinery technology, in contrast to the classical petroleum oil refinery associated with the emission of carbon-based greenhouse gases. As summarized in Table 3, myofibrillar proteins, collagen, gelatin, chitin, chitosan from muscles, viscera, skins, scales, fins or crustacean shell have been demonstrated to satisfy the technological requirements for a novel generation of packaging, named smart packaging, involving interactions between packaging and food or internal packaging atmosphere. In particular, the review has highlighted the great variety of applications of biopolymers from fish industry waste as active packaging, which actively interacts with food and packaging headspace to extend the shelf life of food maintaining nutritional and sensor quality and microbial safety.

Despite the huge industrial potential of fish industry by-products, most of the reviewed applications have a limited Technology Readiness Level (TRL), since most of them have been only validated in the laboratory while the commercial exploitation of biodegradable polymers derived from fish waste is still limited since the functional properties are lower than those of synthetic polymers. The main limitations are brittleness and low mechanical strength, high water solubility, high water vapor permeability. Therefore, the research and development for achieving similar properties of petroleum-based plastics is required while studies on the feasibility at an industrial scale are still missing. Possible routes, analyzed in this review, consist in the formation of composites/blends with several other biopolymers, nanoscale reinforcement, crosslinking and addition of active compounds providing functional properties suitable for active packaging. However, further scientific research is still necessary to better understand the improving mechanisms of material properties at molecular levels analyzing the impact of several factors on the final quality. In addition, a more in-depth understanding of technological aspects of processing, energy balance and costs, environmental emissions and biodegradation conditions are still necessary.

## Figures and Tables

**Table 3:** Biopolymers derived from fish waste: principal applications in food packaging, advantages, disadvantages and strategies for problem resolution.

Biopolymer from Fish Waste	Application	Advantages	Disadvantages	Problem Resolution
**Myofibrillar protein**	edible films	Functional properties with antioxidants, vitamins and coloring agents	Low mechanical properties	Gelatin addition Plasticizer addition
**Marine Collagen**	edible films and coatings	Low cost	Low thermal stability Poor mechanical properties	Blending with biopolymers
**Fish gelatin**	edible films and coatings	Good film-forming properties Low cost Biocompatibility	High hygroscopicity Low barrier properties Low mechanical strength	Cross-linking Nanofillers Blending with biopolymers
**Chitosan**	edible films	Biocompatibility Low cost Antimicrobial properties	Low barrier properties Low mechanical properties	Cross-linking Nanofillers Blending with biopolymers

## Data Availability

The data presented in this study are available on request from the corresponding author.
